# Saccade Adaptation and Visual Uncertainty

**DOI:** 10.3389/fnhum.2016.00227

**Published:** 2016-05-24

**Authors:** David Souto, Karl R. Gegenfurtner, Alexander C. Schütz

**Affiliations:** ^1^Abteilung Allgemeine Psychologie, Justus Liebig Universität GießenGiessen, Germany; ^2^Department of Neuroscience, Psychology, and Behavior, University of LeicesterLeicester, UK; ^3^Allgemeine und Biologische Psychologie, Philipps-Universität MarburgMarburg, Germany

**Keywords:** sensorimotor adaptation, saccade adaptation, saccadic suppression of displacement, visual perception, eye movements

## Abstract

Visual uncertainty may affect saccade adaptation in two complementary ways. First, an ideal adaptor should take into account the reliability of visual information for determining the amount of correction, predicting that increasing visual uncertainty should decrease adaptation rates. We tested this by comparing observers' direction discrimination and adaptation rates in an intra-saccadic-step paradigm. Second, clearly visible target steps may generate a slower adaptation rate since the error can be attributed to an external cause, instead of an internal change in the visuo-motor mapping that needs to be compensated. We tested this prediction by measuring saccade adaptation to different step sizes. Most remarkably, we found little correlation between estimates of visual uncertainty and adaptation rates and no slower adaptation rates with more visible step sizes. Additionally, we show that for low contrast targets backward steps are perceived as stationary after the saccade, but that adaptation rates are independent of contrast. We suggest that the saccadic system uses different position signals for adapting dysmetric saccades and for generating a trans-saccadic stable visual percept, explaining that saccade adaptation is found to be independent of visual uncertainty.

## Introduction

The visuo-motor transformation between a goal in the visual field and a movement plan is plastic, meaning that we can adapt to alterations of the visual feedback, e.g., as after wearing new glasses, or to alterations of the effect of motor commands due to growth, fatigue, injury, or perhaps changes in neural transmission. One of the most studied types of visuo-motor adaption is saccade amplitude adaptation (for a review see Hopp and Fuchs, 2004; Iwamoto and Kaku, 2010; Pélisson et al., 2010; Herman et al., 2013a), which is typically studied with the intra-saccadic step paradigm, in which a saccade target steps during the saccade (McLaughlin, 1967). This shift in visual feedback induces a post-saccadic visual error, or mismatch between the predicted and actual location of the target. This error is corrected gradually, reaching in humans a plateau over the course of some 20–100 saccades.

One major motivation for using intra-saccadic steps to study oculomotor plasticity is that the target step is masked by saccadic suppression of displacement (Stark et al., 1976). One advantage is that if the manipulation is truly invisible, it is also immune to the observer's cognitive strategies. Although cognitive strategies for coping with dysmetria can be interesting in themselves, researchers are more often interested in the gradual and possibly automatic changes of motor commands induced by alterations of visual feedback, as they are possibly more revealing about natural behavior. By the same token, the motor system may be more likely to attribute unseen steps as variability in the motor system and correct the visuo-motor mapping accordingly (e.g., Collins, 2014). Although it is typically assumed that the intra-saccadic step in saccade adaptation paradigms is hardly noticed because of saccadic suppression of displacement, it is not clear whether saccade adaptation is sensitive to the visibility of the error signal. Observers may be uncertain about having seen a step on any given trial and nonetheless detect changes above chance (Morgan et al., 1997). Therefore, the relation between the perception of the intra-saccadic step and adaptation is better conceived in a probabilistic framework.

Recently, two complementary probabilistic ideal adaptor models prescribing how visual uncertainty should affect adaptation have been put forth. We refer to these as the Kalman filter and the contextual relevance models. Burge et al. (2008) manipulated visual uncertainty and showed that an ideal adapter fits reasonably well to manual pointing adaptation data, indicating that visual uncertainty and visuo-motor transformation uncertainty are taken into account in a near optimal way for correcting constant visual errors (see also Wei and Körding, 2010). This prediction is derived from a Kalman filter model, which has proved useful in understanding sensorimotor learning in a range of tasks (Wolpert et al., 1995, 2011; Krakauer and Mazzoni, 2011). In short, the model predicts that the speed at which we should correct for an error should depend on how reliable the visual feedback is relative to the variability of the internal state estimate, which might be derived from an efference copy signal (Wei and Körding, 2010). In particular, the model predicts that a clearly seen visual error should be corrected faster than a poorly seen visual error; conversely a noisier visuo-motor transformation should yield faster corrections, because in both cases the visual feedback is in relative terms more trustworthy.

The Kalman filter model is linear, meaning that the adaptation rate parameter does not depend on error-size, which under some situations is a reasonable assumption (Baddeley et al., 2003). However, recent studies have challenged this model by suggesting that adaptation depends on the relevance of the error to the motor system (Berniker and Kording, 2008, 2011; Wei and Körding, 2009). By a process of causal inference, large errors should provide strong evidence for an external perturbation—such as a target step during a saccade—and therefore adaptation should be slower. Generalization should justify this behavior, since adaptation for a genuine internal perturbation should generate more accurate saccades toward the same location, whereas adaptation to an external event would generate more inaccurate saccades in the absence of this event.

Wei and Körding (2009) found support for the contextual relevance model in a pointing task in which corrections could be estimated based on visual error feedback and proprioceptive information. A Bayesian ideal observer model was fit to the data, essentially depending on the likelihood that the visual error is *relevant* given visual uncertainty (see also Berniker and Kording, 2008, 2011). Interestingly, Wei and Körding (2009) show that a contextual relevance model can fit the results of Robinson et al. (2003) on the effects of error size on the amount (gain) of adaptation achieved in monkeys, increasing linearly for small errors then saturating with large errors.

In the context of saccades, evidence that adaptation-rates depend on causal inference of errors was recently provided by Collins (2014), by showing in a random intra-saccadic step paradigm that the amount of correction from one trial to the other depends on whether the step was correctly detected or discriminated—and therefore was likely to be attributed to an external event—or not. Another indication of the influence of perturbation visibility during the saccade comes from the finding that a gradual adaptation paradigm, in which the step increases across trials, generates a greater extent of adaptation than the classical paradigm, either with adaptation of arm movements or saccades (Wong and Shelhamer, 2011a,b). Because the gradual manipulation is presumably more likely to go unnoticed this could support the role of contextual relevance.

We were interested in contrasting the predictions of the Kalman filter model with the predictions of the contextual relevance model in a saccade adaptation paradigm. In a first experiment we exploited between subject variability to evaluate whether adaptation rates depend on visual uncertainty as predicted by the Kalman filter model. In a second experiment, we tested the effect on adaptation rate of different step sizes, which is a critical test of the contextual relevance model since the Kalman filter model predicts no effect of step size. In particular we expressed step size relative to individual's visual uncertainty, predicting a lower adaptation rate with increased visibility of the step.

To preview on the results we show that none of the models conforms well to the data, which suggests independence of visual signals used to drive saccade adaptation and to integrate position across saccades.

## Methods

### Observers

One author (DS) and 19 undergraduate students from Giessen University (of which 13 were female; mean age of 24 years, ranging from 21 to 35, SD = 4.25 years) participated in the first experiment, 14 in the second experiment (mean age of 26 years, ranging from 18 to 35, SD = 3.45; 12 were female). Eight participated in both experiments. Experiments complied with the principles of the Declaration of Helsinki and were approved by the local ethics committee LEK FB06 at Giessen University (Proposal Number 2013-0020). Written informed consent was obtained prior to the experiment. Students were naïve regarding the purpose of the manipulation.

### Materials

Stimuli were displayed on a 10-bit depth 32-inch Display++ monitor (Cambridge Research Systems, Ltd., Rochester, UK) with a refresh rate of 120 Hz and 1920 × 1080 pixels spatial resolution. The display area subtended 44.4° horizontally and 24.8° vertically at a viewing distance of 90 cm, resulting in 43 pixels per degree. We controlled the stimulus presentation with Matlab (MathWorks, Natick, USA) via the Psychophysics toolbox (Brainard, 1997; Pelli, 1997).

### Experimental procedure

**Figure 1:**
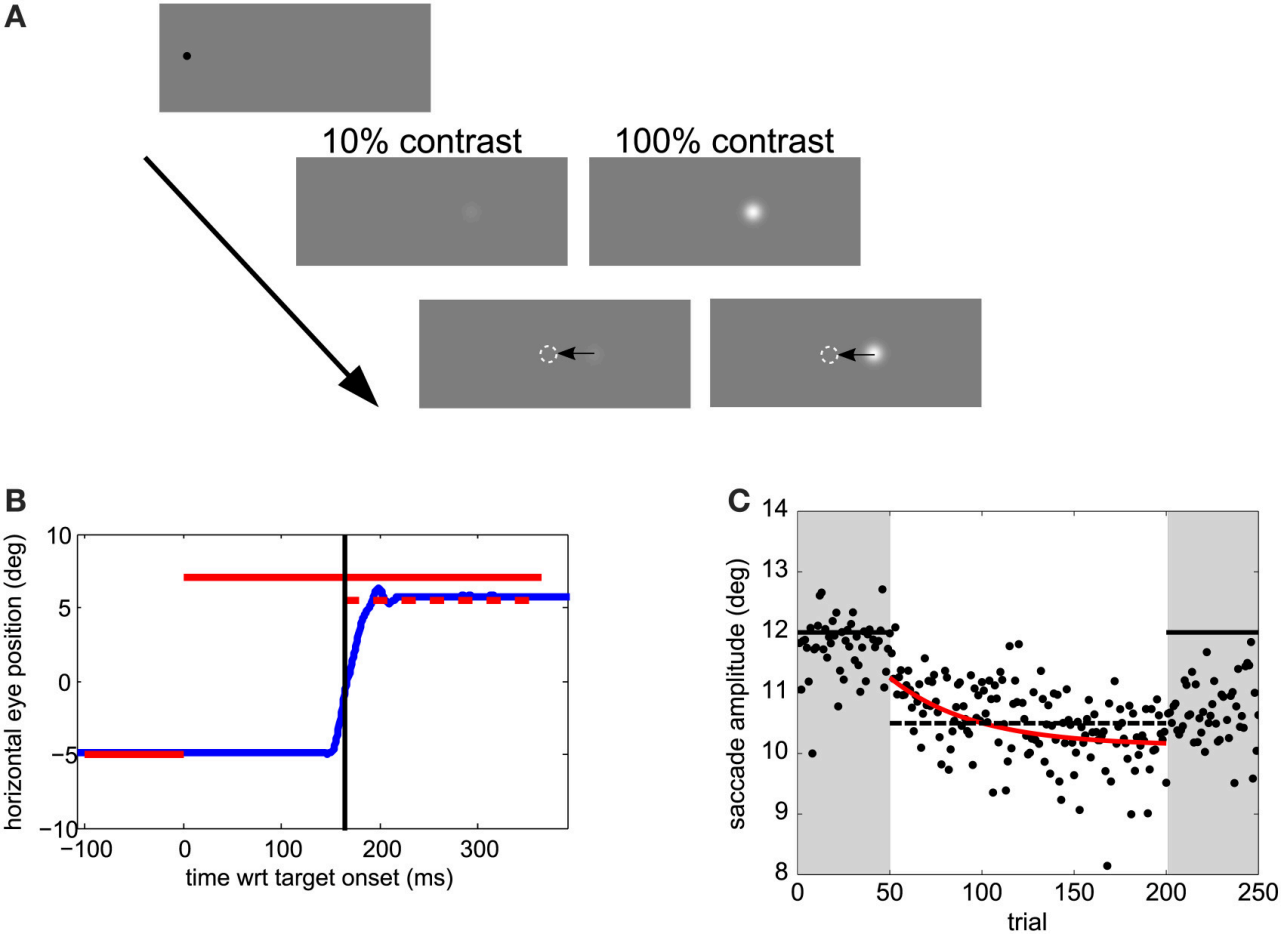
**Experimental methods. (A)** Visual stimuli. Trials started with a black fixation dot at a random location within 0 to −5° (leftward) of the screen center. After a random temporal interval within 0.5–1.5 s, the saccade target appeared 12° rightward from the fixation point. After saccade onset, the target was displaced. **(B)** Time course of one sample trial. Horizontal eye position relative to the screen center as a function of time relative to target onset is shown in blue. The target position and duration during the pre-adaptation and post-adaptation phase is represented by a solid red line. During the adaptation phase the target position is the same before the saccade, but jumps by 1.5° backwards after the saccade—the dashed line in this example. The time at which the target steps is indicated by a vertical black line **(C)** Adaptation data from a sample participant in Experiment 1. Dots show saccade amplitudes in all trials. The best fitting exponential is shown in red. The solid horizontal lines indicate the post-saccadic target position in pre- and post- adaptation phases. The dashed horizontal line indicates the post-saccadic target position in the adaptation phase.

Experiments took place in a dimly lit room. Visual stimuli are shown in Figure 1A. Trials were self-paced. After a key press, a 0.3° black fixation dot was displayed for a random interval between 0.5 and 1.5 s, followed by the presentation of the saccade target 12° rightward of the fixation location. The fixation location was varied between 0 and −5° leftward off the screen center to impede the use of screen edges as a reference. The target was either displaced or displayed at the same location during the saccade for 150 ms. The targets were Gaussian patches of 0.5° standard deviation and had a Weber contrast of 10 or 100%. The background was gray, of luminance 95 cd/m^2^.

In the first experiment, participants took part in two sessions. In each session there was a perception block and adaptation block. The order of perception and adaptation blocks, and of contrast (10% or 100%) in adaptation blocks was balanced across subjects. In the perceptual task observers had to saccade to a target and report whether they saw it step leftward or rightward (2AFC). The next stimulus level was determined by an “updated maximum-likelihood procedure” as implemented in the UML Matlab toolbox (Shen et al., 2015). According to this procedure, on a particular trial, the parameters of a psychometric function (here Weibull) are selected to maximize likelihood. Then, the sweet points for every parameter are calculated. The sweet point of a psychometric function is the point that minimizes expected variance for a particular parameter. An analytical solution for the calculation of sweet points for the slope, threshold and lapse rate is given by Shen and Richards (2012). To go through the different sweet points an up-down procedure is then used. We used a 2-down 1-up rule—i.e., select a lower sweet point (step size) after two correct responses and a larger one after only 1 incorrect response. The sign of the step was randomized. The step size was limited to −4 to 4°. There were 4 perception blocks of 40 trials per session, preceded by 10 training trials, corresponding to 160 trials per psychometric function. Target contrast was alternated by block. No feedback regarding response accuracy was provided.

In the adaptation block (Figures 1B,C) observers' task was only to saccade to the target. As for the perceptual-task blocks they were instructed to saccade toward the target as soon as the target appeared, but not before, while trying to be accurate. In the 50 trials of the pre- and post-adaptation phase the target did not change position. During the 150 trials of the adaptation phase the target stepped backwards of 1.5°, mid-flight during the saccade (cf. Figure 1B).

**Figure 2:**
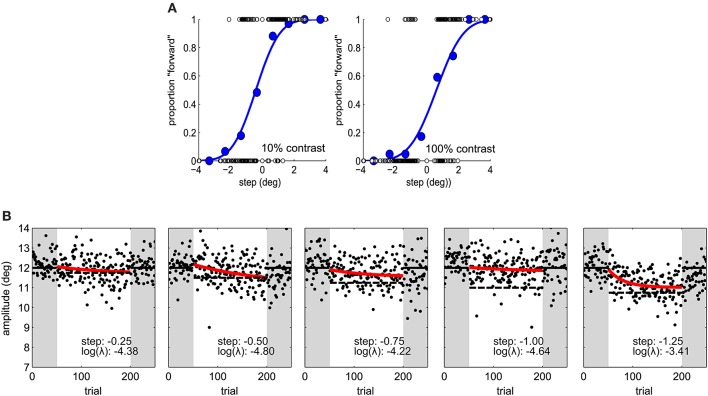
**(A)** Psychometric functions in Experiment 1, for one subject under two contrast conditions. Blue dots represent proportion forward responses with binned step levels for illustration purposes. The presented stimulus levels are indicated by open dots. **(B)** Saccade amplitude in Experiment 2 for one subject for five different step sizes. Same conventions as in Figure 1C.

In the second experiment the same adaptation paradigm was used. Five step sizes (−1.25, −1.00, −0.75, −0.50, and −0.25°) were tested on different sessions and the target was always 100% contrast in the perceptual task and adaptation blocks (Figure 2B). Sessions took place at least 1 day apart. We used a latin-square design for balancing step size transitions across subjects, thus controlling for carry-over effects. In a latin square any condition pair is represented as often as any other and appears only once at a particular session number (Krauth, 2000).

### Eye movement recording

Eye movements were recorded with an Eyelink 1000 (SR-research, Ltd., Osgoode, Canada), video-based eye tracker. The eye tracker was controlled by the Eyelink toolbox port for Matlab (Cornelissen et al., 2002). We did a standard 9-point calibration at the beginning of each perception or adaptation block. For detecting a saccade online, horizontal velocity had to exceed 100 deg/s for two consecutive samples and the eye had to rotate by 3.6° toward the target. This ensured that the target step occurred during the saccade around the time of peak velocity, given saccade durations of about 50–60 ms and a screen update every 8.33 ms.

### Data analysis

We fitted an exponential model to the data with three free parameters: the amplitude of the decay (β), the decay-rate or adaptation rate (λ), and the asymptotic level (α):

(1) $$\begin{array}{r} S\left(t\right)=\alpha +\beta e^{-\lambda t} \end{array}$$

The parameter λ is the adaptation rate parameter and 1/λ corresponds to the exponential time constant. The fit of the adaptation rate parameter was constrained to 0.001 and 1 to avoid infinite values by taking the log of the adaptation rate and very fast adaptation rates, the estimate of which will be very sensitive to error variability in the first trials. We used a nonlinear least-squares fitting procedure to fit the exponential model (OPTI toolbox for Matlab, Currie and Wilson, 2012). The psychometric data were fit with a cumulative Gaussian function using constrained maximum likelihood estimation with Psignifit 3.0 (Fründ et al., 2011).

When correlating adaptation parameters and psychometric function parameters we weighted data by the root mean square error (RMS) of the residuals normalized by total RMS across subjects (*j* index) for that condition (*i* index):

(2) $$\begin{array}{r} w_{ij}=\frac{RMS_{ij}}{\overset{n}{\underset{j=1}{\sum }}RMS_{ij}} \end{array}$$

We used paired *t*-tests for testing for statistical significance, unless there were differences in variability between conditions, in which case we used Wilcoxon signed-rank non-parametric tests.

### Candidate models

A statistically optimal way of correcting errors across trials is to determine the adaptation rate λ by the relative uncertainty of visual information and uncertainty of the state estimate, as obtained by applying a Kalman filter (Kalman, 1960). Hence, the steady-state Kalman gain parameter determining adaptation rate (see Burge et al., 2008; Shadmehr and Mussa-Ivaldi, 2012) is derived from uncertainty of the state estimate σ_*m*_ and of the sensory information σ_*s*_:

(3) $$\begin{array}{r} K=\sigma_{m}^{2}∕\left(\sigma_{m}^{2}+\sigma_{s}^{2}\right) \end{array}$$

The adaptation rate parameter of the exponential describing adaptation is then derived by (see the Appendix for a demonstration):

(4) $$\begin{array}{r} \lambda =-log\left(1-K\right) \end{array}$$

This model makes simplifying assumptions. It supposes a complete exponential decay of the retinal or saccade end-point error, ignoring that adaptation does not asymptote toward zero or even toward the pre-adaptation baseline. Furthermore, several studies indicate that the predicted error rather than retinal error drives adaptation (e.g., Bahcall and Kowler, 2000; Collins and Wallman, 2012; Wong and Shelhamer, 2012).

**Figure 3:**
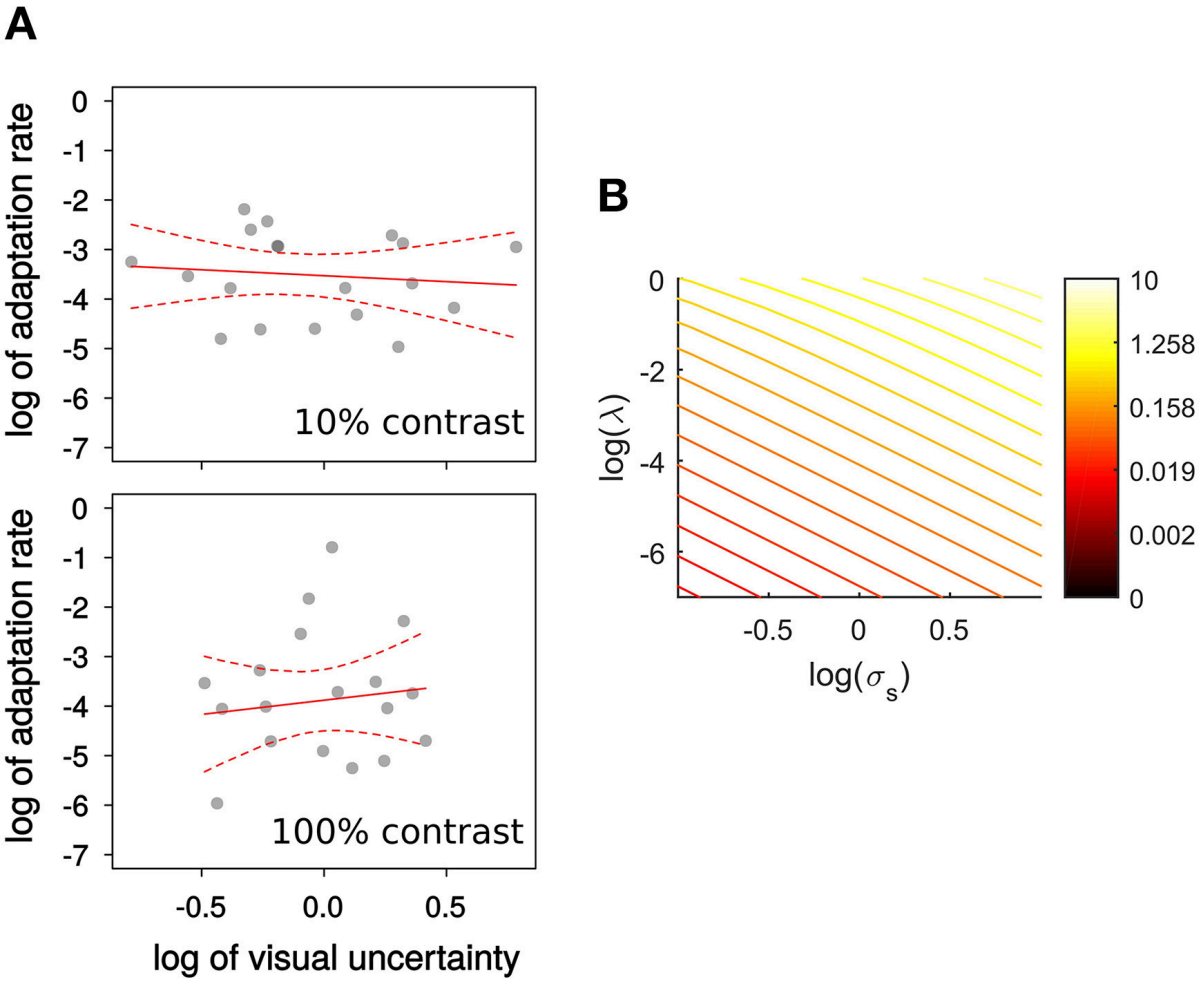
**(A)** Experiment 1, relation between log of visual uncertainty, σ_*s*_, estimated by $\;JND∕\sqrt{2}$, and the log of adaptation rate λ. Observers were tested with 10%, and 100% contrast targets. The red lines represent the best weighted (see text) linear fits. Dashed lines represent the 95% confidence interval for those fits. **(B)** The relation between the logs of visual uncertainty σ_*s*_ and adaptation rate λ is nearly linear for a wide range of visual uncertainties and visuomotor mapping uncertainties σ_*m*_ shown in a color scale.

Figure 3B shows that by applying Equations (3, 4) the log of sensory uncertainty and the log of the adaptation rate are predicted to be in a close to linear (negative) relationship for a wide range of state-uncertainty values. To estimate visual uncertainty σ_*s*_ underlying visual discrimination judgments we calculated the just-noticeable-difference (JND) as the difference between the step size yielding 84% correct and the one yielding 50% correct. Then $\sigma_{s}=JND∕\sqrt{2}$ (Ernst et al., 2004; Burge et al., 2008). One of the observers' psychometric functions were flat within the range of steps displayed, and therefore their data is not included. In another participant this was only the case for the 100% contrast condition.

As a measure of the proportion of adaptation we took the difference between the median amplitude over 50 trials in the pre-adaptation phase and over 50 trials at the end of the adaptation phase, divided by the step size. The Δs parameter—or starting point—corresponds to the difference between the amplitude in the pre-adaptation phase and the amplitude at which the fitted exponential starts. The Δs parameter informs about rapid learning, indicative of the use of cognitive strategies, which might be more prevalent with more visible steps. This parameter should hover around zero if participants do not behave differently when steps exceed perceptual threshold.

## Results

In two experiments we tested observers' perceptual thresholds and adaptation rates, asking how visual uncertainty relates to saccade amplitude adaptation. In a first experiment we varied the target contrast between subjects. In a second experiment we varied step size between subjects. Individual data, including untransformed adaptation rates and thresholds, can be found in Tables 1, 2.

**Table 1:** **Individual data in Experiment 1 showing original and log-transformed values**.

	**JND**		**log(*****JND*****/**√2)		**PSE**		λ		**log (**λ**)**		Δ ***s***		**Proportion change**	
	**Contrast**		**Contrast**		**Contrast**		**Contrast**		**Contrast**		**Contrast**		**Contrast**	
**Obs**.	**10%**	**100%**	**10%**	**100%**	**10%**	**100%**	**10%**	**100%**	**10%**	**100%**	**10%**	**100%**	**10%**	**100%**
1	0.74	0.55	−0.28	−0.41	−0.90	−0.77	0.010	0.079	−4.61	−2.54	0.15	0.43	0.74	0.55
2	0.54	0.61	−0.42	−0.36	−1.14	−0.98	0.039	0.003	−3.25	−5.96	0.07	0.71	0.54	0.61
3	0.60	0.94	−0.37	−0.18	−0.75	−0.25	0.029	0.017	−3.54	−4.06	0.11	0.49	0.60	0.94
4	1.40	0.99	0.00	−0.16	−0.12	0.52	0.025	0.030	−3.68	−3.51	0.43	0.13	1.40	0.99
5	0.74	0.67	−0.28	−0.32	−0.58	0.60	0.053	0.161	−2.93	−1.83	0.17	−0.35	0.74	0.67
6	1.48	1.08	0.02	−0.12	−1.63	−1.13	0.007	0.009	−4.97	−4.71	0.72	0.52	1.48	1.08
7	0.68	0.97	−0.32	−0.16	−0.83	0.12	0.112	0.018	−2.19	−4.04	−0.35	0.59	0.68	0.97
8	0.75	1.35	−0.28	−0.02	−0.63	−0.71	0.066	0.006	−2.71	−5.11	0.15	0.52	0.75	1.35
9	0.57	0.89	−0.39	−0.20	−0.69	−0.24	0.074	0.029	−2.60	−3.53	−0.01	0.16	0.57	0.89
10	2.07	1.75	0.17	0.09	−1.25	0.32	0.015	0.009	−4.18	−4.70	1.30	0.56	2.07	1.75
11	1.31	0.69	−0.03	−0.31	−0.92	−0.32	0.008	0.018	−4.80	−4.01	−0.06	−0.03	1.31	0.69
12	1.15	1.26	−0.09	−0.05	−0.57	0.34	0.023	0.005	−3.78	−5.25	0.62	0.85	1.15	1.26
13	0.56	0.45	−0.40	−0.50	−1.96	−	0.052	0.149	−2.95	−1.91	0.24	−0.29	0.56	0.45
14	1.12	0.70	−0.10	−0.31	−0.94	−0.67	0.023	0.453	−3.78	−0.79	−0.18	0.19	1.12	0.70
15	0.82	0.60	−0.24	−0.38	−0.62	−0.65	0.056	0.102	−2.87	−2.28	0.37	−0.09	0.82	0.60
16	0.95	0.80	−0.17	−0.25	−0.92	−0.13	0.053	0.038	−2.93	−3.28	−0.25	0.20	0.95	0.80
17	0.82	1.49	−0.24	0.02	−0.88	−0.89	0.088	0.007	−2.43	−4.91	0.11	0.55	0.82	1.49
18	0.82	0.47	−0.24	−0.48	−1.51	−0.91	0.013	0.024	−4.31	−3.74	0.36	−0.01	0.82	0.47
19	0.94	0.76	−0.18	−0.27	−0.74	0.07	0.010	0.024	−4.60	−3.72	0.16	0.47	0.94	0.76
Mean	0.95	0.90	−0.20	−0.23	−0.92	−0.32	0.040	0.062	−3.53	−3.68	0.22	0.30	0.95	0.90
SD	0.38	0.35	0.16	0.16	0.42	0.53	0.03	0.10	0.84	1.30	0.37	0.33	0.38	0.35

JND, just noticeable difference (85% threshold); PSE, point of subjective equality; λ, adaptation rate; Δs, difference between the exponential fit starting point amplitude and pre-adaptation median amplitude; proportion change, refers to the amplitude change between pre-and end of adaptation trials normalized by the size of the step.

**Table 2:** **Individual data in Experiment 2 showing original and log-transformed values**.

				λ					**log (**λ**)**					Δ***s***					**Proportion change**				
				**Step size**					**Step size**					**Step size**					**Step size**				
**Obs**.	**JND**	**log(*JND*/√2)**	**PSE**	**−0.25**	**−0. 5**	**−0.75**	**−1.00**	**−1.25**	**−0.25**	**−0. 5**	**−0.75**	**−1.00**	**−1.25**	**−0.25**	**−0. 5**	**−0.75**	**−1.00**	**−1.25**	**−0.25**	**−0. 5**	**−0.75**	**−1.00**	**−1.25**
1	0.90	-0.20	0.32	0.013	0.008	0.015	0.10	0.033	-4.38	-4.8	-4.22	-4.64	-3.41	0.12	-0.04	0.37	0.46	0.22	1.81	1.93	0.97	0.66	0.89
2	0.54	-0.42	-0.49	0.023	0.019	0.178	0.336	0.453	-3.77	-3.96	-1.73	-1.09	-0.79	-0.08	-0.05	0.10	-0.62	0.14	0.00	0.76	0.95	0.44	0.46
3	1.96	0.14	-0.51	0.401	0.032	0.009	0.007	0.040	-0.91	-3.43	-4.67	-4.96	-3.22	0.00	-0.19	-0.08	0.49	0.23	0.00	1.26	0.84	1.11	0.79
4	1.02	-0.14	-0.02	0.001	0.010	0.009	0.022	0.017	-6.91	-4.64	-4.72	-3.82	-4.08	0.32	0.12	0.44	0.25	0.53	1.51	0.96	0.80	0.55	0.67
5	1.63	0.06	-1.30	0.001	0.158	0.032	0.053	0.566	-6.91	-1.84	-3.45	-2.94	-0.57	0.13	-0.06	-0.02	0.02	-0.20	0.69	0.55	0.25	0.25	0.39
6	0.97	-0.17	-0.58	0.109	0.040	0.007	0.57	0.030	-2.21	-3.23	-4.91	-2.87	-3.49	-0.11	-0.09	0.10	0.04	0.14	1.24	1.04	1.84	0.51	0.95
7	0.83	-0.23	-0.44	0.010	0.105	0.067	0.097	0.008	-4.63	-2.25	-2.7	-2.33	-4.81	-0.04	0.09	-0.04	-0.21	0.19	0.68	1.07	0.5	0.54	1.31
8	2.12	0.18	-1.20	0.007	0.990	0.045	0.107	0.131	-4.91	-0.01	-3.09	-2.24	-2.03	-0.07	0.11	0.2	-0.12	-0.26	0.03	0.21	0.47	0.55	0.61
9	2.74	0.29	-1.41	0.008	0.013	0.016	0.261	0.038	-4.78	-4.31	-4.14	-1.34	-3.28	0.39	0.16	0.36	-0.24	0.19	3.55	0.46	0.92	0.68	0.93
10	1.07	-0.12	-0.83	0.027	0.026	0.009	0.027	−	-3.59	-3.65	-4.74	-3.61	−	0.11	0.10	0.26	0.40	−	1.77	1.06	1.75	0.79	−
11	1.18	-0.08	-0.28	0.018	0.779	0.17	0.007	0.015	-4.04	-0.25	-4.05	-4.91	-4.17	0.13	-0.49	0.11	0.77	0.27	3.77	0.97	1.05	1.60	0.76
12	2.87	0.31	-1.28	0.033	0.115	0.180	0.058	0.006	-3.41	-2.16	-1.71	-2.85	-5.14	0.61	0.39	0.41	0.42	0.46	2.43	0.79	0.98	0.82	1.18
13	2.39	0.23	-0.91	0.020	0.016	0.008	0.018	0.024	-3.89	-4.12	-4.86	-4.00	-3.73	0.31	0.25	0.32	0.58	0.48	4.26	1.03	1.84	1.58	1.03
14	1.97	0.14	-0.91	0.143	0.007	0.006	0.001	0.007	-1.95	-4.94	-5.09	-6.91	-4.92	0.21	0.05	0.71	0.69	0.20	0.84	1.19	2.28	0.77	1.84
Mean	1.59	0.00	-0.70	0.058	0.166	0.043	0.076	0.105	-4.02	-3.11	-3.86	-3.46	-3.36	0.15	0.03	0.23	0.21	0.20	1.61	0.95	1.10	0.78	0.91
SD	0.73	0.21	0.49	0.103	0.300	0.058	0.097	0.176	1.61	1.54	1.11	1.51	1.39	0.20	0.20	0.21	0.39	0.22	1.37	0.39	0.58	0.39	0.37

See Table 1 legend.

### Adaptation rate and JND

**Figure 4:**
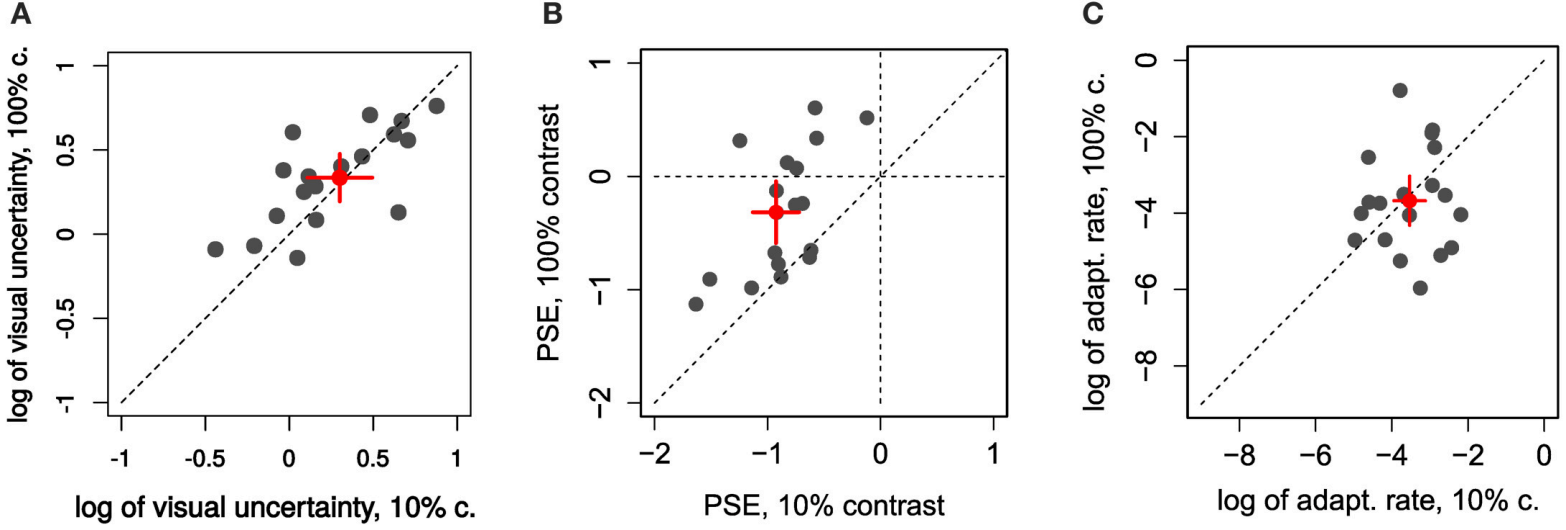
**Individual comparison of 10% and 100% contrast conditions in Experiment 1 for: (A) the log of visual uncertainty (log of $\;JND∕\sqrt{2}$) (B) PSE and (C) log of adaptation rate**. Red dots and error bars represent averages and 95% confidence intervals.

In the first experiment we aimed to manipulate visual JNDs by having 10% and 100% contrast targets (Zimmermann et al., 2013). Figure 2A and Figures 4A,B compares PSEs and JNDs of the psychometric functions obtained from the step direction discrimination task for low and high contrasts in individual subjects. On average, PSEs are shifted backwards by −0.9° (SD ± 0.43) with a low contrast, whereas PSEs for high contrast targets are nearer to veridical, −0.31° (SD ± 1.1). Between-subjects variance was more than twice as large for the 100% contrast targets compared to 10% contrast targets (0.13 and 0.30, respectively), Levene's test, *F*_(1, 34)_ = 6.163, *p* < 0.018. The effect of contrast on PSEs was statistically significant, Wilcoxon signed-rank test *W*_(17)_ = 6, *p* < 0.0001. However, the JNDs were similar with the 10% (1.46 ± 0.62) and 100% (1.37 ± 0.40) contrast targets and not statistically different from each other, *t*_(17)_ = 1.008, *p* = 0.328, as shown in Figure 4A. Although our explicit manipulation of visual uncertainty was not successful, we can compare visual uncertainty and adaptation rates across observers.

Learning-rates (in log units, Figure 4C) were on average −3.67 in log units (SD ±1.09). In more relatable terms, this corresponds to an exponential time constant of about 39 trials (1/λ)—i.e., the time it takes the function to reach about a third of the initial value—going from some 14 to 106 trials (±1 log unit).

In Figure 3 we show the relation between log of adaptation rate λ and the log of $\;JND∕\sqrt{2}$ as an estimate of visual uncertainty, σ_*s*_ (Burge et al., 2008), in relation to the predictions of a Kalman filter model (Figure 3B). The red line represents the least-squares fit, weighted by a function of RMS of residuals. For both contrasts the relation is flatter than predicted. In particular, the slope predicted by the integration model (of −2: an increase in 1 log unit of visual uncertainty reduces λ by 2 log units) is outside the 95% confidence interval for the slope of the weighted linear regression for the low contrast (−0.23, CI: −1.33 to 0.85) and high contrast targets (0.94, CI: −1.60 to 2.74). Both intervals contain zero^1^. Niemeier et al. (2003) showed that saccade endpoint variability is positively correlated with the amount of saccadic suppression of displacement, and proposed that endpoint variability indicates uncertainty of visual and extra-retinal signals necessary to maintain spatial constancy after the saccade. Visual and motor uncertainty should affect adaptation rates according to the Kalman filter model, but we do not know to what extent saccade endpoints reflect one or the other. Nonetheless, to know whether there is a relationship between visual uncertainty and learning unmediated by saccade endpoint variability we calculated the partial correlation between the log of λ and the log of visual uncertainty, controlling for endpoint variability. This correlation was not significant (all *p* > 0.58) and the slope estimate was again near to 0 (low contrast: 0.14; high contrast: −0.09). However, we confirmed the findings of Niemeier et al. (2003), that is a correlation between endpoint variability and log of visual uncertainty (σ_*s*_) for low contrast, *r*_(17)_ = 0.58, *t*_(17)_ = 2.922, *p* = 0.009, and high contrast targets, *r*_(16)_ = 0.66, *t*_(16)_ = 3.573, *p* = 0.003.

### Adaptation rate and step size

**Figure 5:**
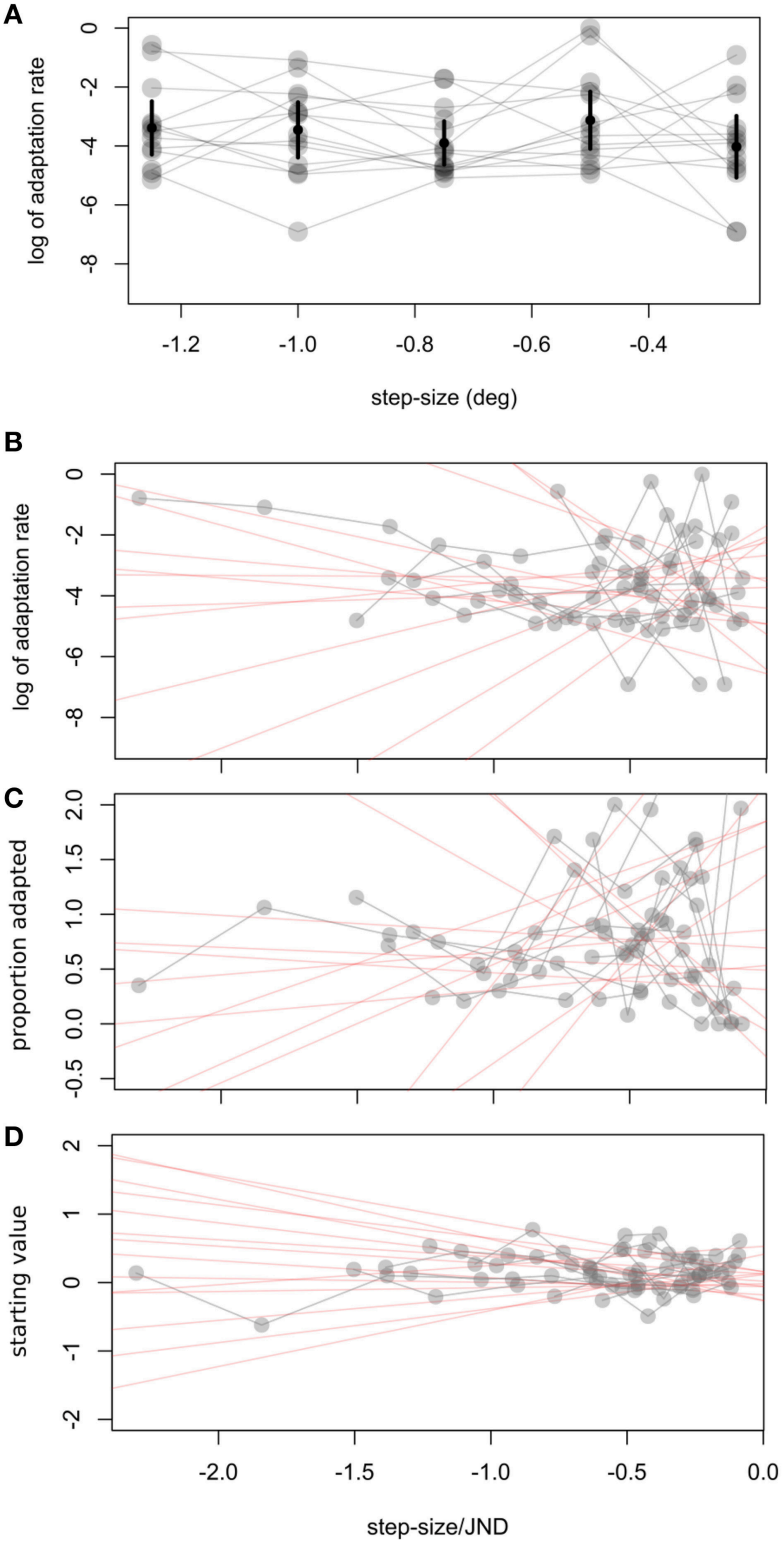
**Experiment 2, relation between step size and adaptation parameters. (A)** Relation between step size and log of adaptation rate λ. Error bars represent the weighted (see text) average and confidence interval for every step. To take into account individual JNDs we show in **(B–D)** how different fit parameters are related to the ratio of step size to JND. Δs refers to the difference between the starting point measured by the pre- adaptation median and the starting point determined by the exponential fit. In **(B–D)** we fit a weighted linear function to individual data, giving the red shaded lines. Every subject's data is connected by a gray line.

In a second experiment we calculated observers' visual JNDs and learning rates for different step sizes (0.25–1.5°). Adaptation rates were on average −3.53 in log units (SD ± 1.49). Figure 2B shows a great deal of variability in learning rates within the same observer. Figure 5 shows how adaptation parameters depend on step size. The ratio between step size and individual JND gives a measure of how likely an observer is to see the target jump. Therefore, we calculated linear regressions to individual data to test whether adaptation rate is lower for more visible target steps—as predicted by the contextual relevance model—or the same—as predicted by the Kalman filter model or by a model assuming independent position signals for perception and oculomotor control.

Adaptation rates were similar across step size, as tested with a one-way ANOVA, *F*_(4, 55)_ = 1, *p* = 0.416 (Figure 5A). Variability for other parameters was also very high, especially for the percent adaptation measure (Figure 5C)^2^. Other parameters tested were the starting point of the exponential (Δs) and the proportion adapted. A *t*-test on the slope of the individual linear regressions between step size/JND and adaptation parameters showed that none were significantly different from zero (p*s* > 0.14; 95% C.I.: log of adaptation rate: −2.5 to 1.9; Δs: −0.44 to 0.14, proportion adapted: −0.58 to 1.33). Untransformed adaptation rates (λ) were also independent of step size (0.002, C.I: −0.12 to 0.12) or step-size/JND (0.043, C.I.: −0.13 to 0.21).

## Discussion

We tested the influence of visual uncertainty and error-size on saccade adaptation parameters. Visual uncertainty was evaluated in a separate block in which observers had to discriminate the direction of the step. We found that:

1. The point of subjective stationarity is shifted to inward steps in low contrast targets compared to high contrast targets.
2. Adaptation rate is unrelated to visual uncertainty (JNDs) across subjects.
3. Adaptation parameters are unrelated to error-size manipulated within subjects.

We observed a shift in PSEs for low-contrast targets: some backward changes in position were perceived as stationary and no change in position was perceived as forward step. PSEs for high contrast targets were closer to zero such that judgments were more veridical. Despite these large differences in perceived stationarity, there were no systematic differences in the adaptation rates in the two contrast conditions. This is clear evidence for a dissociation between perception and adaptation. The origin of this shift in PSEs for low contrast targets is unclear. One explanation could be that for low contrast targets, there is a stronger reliance on prior knowledge that visible targets are likely to be presented near the fovea. If the eccentricity is underestimated, a backward step should be perceived as stationary, and no step perceived as a forward step. If error-correction was based on this information too, we should have observed a difference in the amount of adaptation, between low and high contrast targets, which was not the case. A foveal localization bias in the relative localization of peripheral stimuli has been observed before. It was shown to increase with shorter presentation times and lowered discrimination thresholds, consistent with the reliance on prior knowledge (Müsseler et al., 1999; Brenner et al., 2008). Incidentally, we did not find the effect of contrast on JNDs that we expected. Zimmermann et al. (2013) showed slightly higher sensitivity to displacements in 97% compared to 10% contrast targets, but they used rather different stimuli, differing in polarity and spatial frequency content. A manipulation of post-saccadic error timing might prove as a more robust way of manipulating adaptation (Shafer et al., 2000) and visual uncertainty within participants (e.g., Zimmermann et al., 2013) in the future.

The contextual relevance model (e.g., Wei and Körding, 2009; Collins, 2014) makes testable predictions, especially when comparing the effect of different step sizes on adaptation rates, since the Kalman filter model predicts that adaptation rates are independent of step size. The contextual relevance model supposes that the amount of adaptation is proportional to the likelihood that an event is relevant to the motor system given sensory data. This likelihood is higher for smaller steps and higher sensory uncertainty (Wei and Körding, 2009). An extreme case would be that this likelihood is estimated to be close to one, either because visual uncertainty is very high (i.e., low visual-to-noise ratio for the sensory information) or because prior evidence for an external perturbation is very low, or a combination of the two. In this case adaptation rates should follow the predictions of the Kalman filter model. However, when uncertainty is small and steps get larger we should observe less adaptation as the likelihood of being relevant gets lower. Therefore, according to this model the relationship between target location uncertainty and the log of the adaptation rate parameter should be flatter than predicted by the Kalman filter model, or even be in a positive relationship as there should be no adaptation at all when there is certainty that the error signal is irrelevant to the motor system. In the second experiment, we should see that adaptation rates decrease with step size.

Our results show that the Kalman filter model does not explain saccadic adaptation rate when estimating visual uncertainty by step discrimination. If anything, we observed slightly faster learning rates with more visible targets. The results cannot be explained by the effect of contextual relevance either, since error-size had no effect on adaptation rates. It remains a possibility that larger errors would show a non-linear relationship (see Wei and Körding, 2009; Collins, 2014). However, with the range of steps we used, from 0.1 to up to 2.5 times the JND values, we should expect attribution to an external event with certainty for the larger steps and therefore observe little adaptation. We do not question that an effect of step size could be found with even larger steps, as it was shown by others (Collins, 2014). However, the results from Herman et al. (2013b) are clearly contradicting ours as they found that adaptation rates strongly depend on step size in a similar range of step sizes to ours. We think that the difference between our results and the results by Herman et al. is due to different fitting procedures. While Herman et al. used an exponential fit with only two free parameters for the amplitude and rate of adaptation, we have an additional parameter for the asymptotic value. Without this additional parameter, adaptation rate also reflects differences in the asymptotic value of adaptation, such that it artificially scales with step-size. As the exponential is not allowed to plateau and therefore to fit the data at the end of the adaptation phase, the best fitting function is one having a very low adaptation rate parameter that scales to the adapted amplitude—and therefore step size. With a high adaptation rate parameter the function would reach zero before the end of the adaptation trials. Accordingly, Herman et al. adaptation rate estimates are two orders of magnitude smaller than ours and those reported in the literature (e.g., Srimal et al., 2008). Therefore, their study can inform about whether adaptation took place with different steps, but not about differences in adaptation rates. Nonetheless, Herman et al. found that the proportion of adaptation, the ratio of adaptation to step-size, is fairly constant across step-sizes, which is consistent with our results.

Our results may appear to be also directly at odds with those of Collins (2014), showing smaller corrections from one trial to the other when a step was correctly detected. In that study, random steps were applied during the saccade. Although it is unclear whether random-adaptation paradigms imply similar mechanisms to adaption with constant steps, Srimal et al. (2008) found similar adaptation rates under both paradigms. The method used by Collins (2014) may have been more sensitive in detecting small changes in adaptation rate, however, a large effect of step size was also found. This may not necessarily be understood in terms of causal attribution. An alternative explanation can be that it is the size of the error that matters in the process of binding the post-saccadic target location to the expected target location, instead of the likelihood of the error being relevant to the motor system. Detection of the step in a random adaptation paradigm is necessarily positively correlated with the size of the step, and therefore the finding that the amount of adaptation on the next trial depends on the target being detected could be mediated by the effect of step size. For instance, adaptation could be slower if the error is larger than a critical value. Further research would be needed to examine a *critical error* hypothesis as opposed to *contextual relevance* defined by the computation of likelihoods. Spatial attention could be such an error binding mechanism. The critical error would be determined by the size of the attention focus. Accordingly, saccade adaptation has been shown to be modulated by attentional load (Gerardin et al., 2015).

An interesting suggestion of our results is that spatial constancy across saccades, our ability to judge the displacement of a target across a saccade, does not involve the same visual signals as those driving saccade adaptation. Collins (2014) arrived to a similar conclusion by noting substantial saccade adaptation for target steps that were not correctly detected or discriminated. By showing that visual uncertainty and PSE are poorly related to saccade adaptation we suggest that there is an even deeper dissociation. Then, in what way is the use of visual information for oculomotor adaptation and perception different? A recent study, showing saccade adaptation for steps presented during the saccade (Panouillères et al., 2013), suggests a much earlier time-window over which visual signals can drive adaptation than previously known. Perceptual judgments might include information acquired over a larger time-window, potentially explaining a lack of correlation between perceptual performance and motor adaptation.

Movement variability (i.e., saccade endpoints) has been shown to be strongly related to suppression of displacement (Niemeier et al., 2003). However, we found that movement variability did not mediate adaptation rate. Given that both eye state uncertainty and visual uncertainty should contribute to saccade endpoint variability, this seems surprising. However, if we assume that a large part of saccade endpoint variation has a perceptual origin (van Beers, 2007), not only is a large correlation between movement variability and saccadic suppression of displacement unsurprising, but also a lack of correlation between movement variability and adaptation rate, since it should be driven by the uncertainty about the state of the eye, e.g., as derived from an efference copy signal. Collins et al. (2009) showed that the discrimination of intrasaccadic steps is independent of saccade landing position, indicating that the efference copy is accurate, even though the target is perceptually remapped after saccade adaptation.

A last point is that there is no indication that the observers use cognitive strategies when they are aware of the manipulation. For instance, the subject could target a different location than the target location knowing that the target would step. Interestingly we show no evidence that perception of the step matters for measuring saccade adaptation, as we see no trace of faster adaptation rates for clearly visible targets, especially looking at the presence of an immediate error-correction at the beginning of the adaptation phase. Overall, correction is gradual and independent of step visibility. This concurs with hand movement studies showing that explicit strategies hardly overrule implicit corrections (Mazzoni and Krakauer, 2006). Similarly, we showed that even when observers are instructed to process information at a specific location within a target object they still show gradual saccadic adaptation (Schütz et al., 2014; Schütz and Souto, 2015).

In conclusion, saccadic suppression of displacement and saccade adaptation may rely on signals that are independent. This strengthens the reported observation that saccade adaptation occurs even when observers are unaware of the intra-saccadic step. We propose that the detection of mismatches across saccades and oculomotor control rely on different signals. On the one hand the detection of intra-saccadic steps requires a comparison of pre- and post-saccadic position signals which could be hampered by trans-saccadic integration. On the other hand saccadic adaptation requires the comparison of post-saccadic error to a prediction of the saccade endpoint given the target goal, this mismatch with the actual landing position may be used to modify the sensorimotor transformation. A fundamental difference between our results and those obtained by Burge et al. (2008) with pointing movements is that the hand movement does not modify the visual input, whereas vision across saccades requires an efference copy signal to recover position in spatial coordinates (e.g., Collins, 2014). Experiencing a stable visual world across saccades requires a conservative bias, whereas the oculomotor system benefits from keeping sensitive to spatial errors. Finally, there was no sign of the use of cognitive strategies neither when the target was well over perceptual threshold nor when it was not, which is good news for the use of the saccadic adaptation paradigm.

## Author contributions

DS, KRG, and ACS contributed to the conception, research design and interpretation of the experiments. DS and ACS wrote the final manuscript. DS analyzed the data.

### Conflict of interest statement

The authors declare that the research was conducted in the absence of any commercial or financial relationships that could be construed as a potential conflict of interest.

## Footnotes

^1^A regression model supposes that there is no error in estimating visual uncertainty. However, we also tested an orthogonal regression model. The slope was obtained by doing a principal component analysis on the log of adaptation rate against the log of visual uncertainty. The slope of the first component is the one that minimizes the orthogonal error. Confidence intervals (95% percentiles) for the slope were obtained by bootstrapping (Efron and Tibshirani, 1994): −0.11 to 0.10 for 10% contrast targets and −0.06 to 0.07 for 100% contrast targets.

^2^Instead of using the raw data to estimate proportion adapted we also used the parameter β (scale) of the best fitting exponential and calculate its ratio to step size. However, the fitted slopes were just as variable with this measure.

## Appendix

Here we give a demonstration for Equation (4), λ = −*log*(1 − *K*), relating the exponential time constant λ and the Kalman filter constant *K* (Equation 4 of Burge et al., 2008), as we failed to find a clear demonstration elsewhere. *K* represents the amount of error on the previous trial that is corrected on the current trial (Equation 3 of Burge et al., 2008), *x*(*t*) is the saccade amplitude on trial *t*:

(A1) $$\begin{array}{l} x\left(t+1\right)=x\left(t\right)+K\varepsilon \left(t\right) \end{array}$$

Error is simply the target amplitude *T* minus saccade amplitude:

(A2) $$\begin{array}{l} \varepsilon \left(t\right)=T-x\left(t\right) \end{array}$$

As error is known to decay exponentially, we have that:

(A3) $$\begin{array}{l} \varepsilon \left(t\right)=e^{-\lambda t} \end{array}$$

From Equations (A1) and (A2), it follows that:

(A4) $$\begin{array}{l} \varepsilon \left(t\right)-\varepsilon \left(t+1\right)=x\left(t+1\right)-x\left(t\right)=K\varepsilon \left(t\right) \end{array}$$

Given Equation (A3) this can be rewritten as:

(A5) $$\begin{array}{l} e^{-\lambda t}-e^{-\lambda \left(t+1\right)}=e^{-\lambda t}-e^{-\lambda t}e^{-\lambda }=e^{-\lambda t}\left(1-e^{-\lambda }\right) \end{array}$$

Therefore, using the identity in Equation (A4):

(A6) $$\begin{array}{l} Ke^{-\lambda t}=e^{-\lambda t}\left(1-e^{-\lambda }\right) \end{array}$$

Meaning *K* = 1 − *e*^−λ^ or *e*^−λ^ = 1 − *K*. Taking −*log* on both sides gives λ = −*log*(1 − *K*).
